# Ultrasound-assisted synthesis of graphene@MXene hybrid: A novel and promising material for electrochemical sensing

**DOI:** 10.1016/j.ultsonch.2022.106208

**Published:** 2022-10-22

**Authors:** Shenchao Shi, Ruizheng Zhong, Lele Li, Chidan Wan, Can Wu

**Affiliations:** a Department of Hepatobiliary Surgery, Union Hospital, Tongji Medical College, Huazhong University of Science and Technology, Wuhan 430022, China; b Collaborative Innovation Center for Advanced Organic Chemical Materials Co-constructed by the Province and Ministry, Ministry of Education Key Laboratory for the Synthesis and Application of Organic Functional Molecules, School of Materials Science & Engineering, Hubei University, Wuhan 430062, China

**Keywords:** Ultrasound-assisted synthesis, Liquid-phase exfoliation, MXene, Graphene, Electrochemical sensors

## Abstract

To date, multiple graphene@MXene hybrids have been reported *via* various synthesis approaches, but almost all the graphene@MXene hybrids inevitably used the reduced graphene oxide that prepared by chemical oxidation/reduction method, which generally involved the complex and dangerous operation procedure, and the highly toxic chemical reagent. How to prepare graphene@MXene hybrid through a simple, safe and eco-friendly synthetic route is highly desired. Compared with traditional synthesis technology, ultrasound synthesis strategy displays the merits of simplicity, low cost and environment protection. Herein, MXene (Ti_3_C_2_T_x_) nanoflakes coupled with graphene nanosheets (graphene@MXene) were prepared in *N*-methylpyrrolidone (NMP) by simple ultrasound-assisted liquid-phase exfoliation method for the first time. Besides, the effect of types of solvent with different viscocity, sonication temperature and sonication duration time on the property of graphene@MXene hybrids were systematacially investigated. It is found the liquid-phase exfoliated graphene owned excellent electron transfer ability and the MXene (Ti_3_C_2_T_x_) nanoflakes possessed outstanding adsorption property, the as-synthesized graphene@MXene hybrid exhibited significant signal synergistic enhancement effect toward the oxidation of hazardous veterinary drug residue compound (chlorpromazine) and food additives (rhodamine B). Based on this, a novel and sensitive electrochemical sensor was fabricated, the linear detection ranges were 5 nM to 0.5 μM for chlorpromazine with sensitivity of 1090 µA μM^−1^ cm^−2^, and 10 nM to 2.5 μM for rhodamine B with sensitivity of 440 and 102.14 µA μM^−1^ cm^−2^. Besides, the detection limits were evaluated to be as low as 1.25 nM and 2.45 nM for chlorpromazine and rhodamine B, respectively.

## Highlights

• Two-dimensional graphene@MXene nanohybrid was facilely prepared through ultrasonic liquid-phase exfoliation.
• In-situ ultrasound-assisted synthesis of graphene@MXene hybrid suspension exhibited high stability.
• Significant synergistic signal enhancement effect was displayed due to the integration of graphene and MXene.
• Novel electrochemical sensing platform for the sensitive detection of chlorpromazine and rhodamine B was developed.

## Graphical abstract

**Figure:**
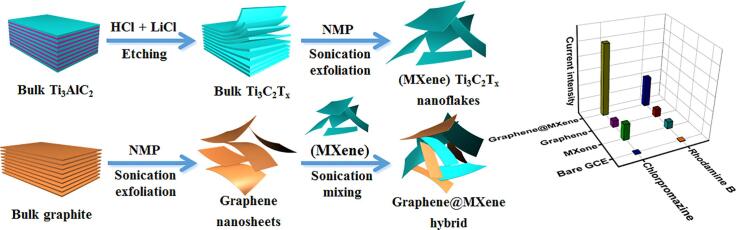

## 1 Introduction

Two-dimensional (2D) materials, including graphene, black phosphorus (BP), transition metal dichalcogenides (TMDs), layered double hydroxides (LDHs), transition metal carbides and nitrides (MXenes) have attracted extensive attention of the scientific community for their unique chemical and physical property [1], [2], [3], [4], [5], [6], [7]. Among these 2D materials, MXenes have aroused huge research upsurge in terms of the synthesis and application since its first discovery in 2011 by Yury Gogotsi [8], [9], [10], [11]. In general, MXenes are prepared from its layered ternary MAX phase by selective etching the A elements (group III or IV), and the obtained products were denoted as M_n+1_X_n_T_x_, where M represents Ti, V, Nb, and Mo, x represents C and/or N, n = 1, 2, or 3, and T_x_ stands for the surface functional group (OH, O, and/or F) [12]. Benefiting from its metal-like electrical conductivity, abundant surface groups and superior mechanical flexibility, MXenes have displayed excellent electrochemical performance and been widely applied in the field of electrochemical sensors [13], [14]. For examples, MXene (Ti_3_C_2_T_x_) nanoflakes modified glassy carbon electrode was successfully used to simultaneously monitor environmental pollutants 4-chlorophenol and 4-nitrophenol with high detection sensitivity [15]. MXene (Ti_3_C_2_T_x_) nanosheets modified screen-printed carbon electrode displayed excellent signal enhancement effect toward the oxidation and sensing of acetaminophen and isoniazid [16]. In order to further enhance the electrochemical property and dig the sensing application potential of MXene-based materials, integrating other functional materials, including noble metal nanoparticles, metal oxide, porous carbon and so on with MXene has been demonstrated to be a feasible way [17], [18], [19].

Among these functional materials, graphene is no doubt a type of ideal additive because of its outstanding electrochemical sensing property. For instance, the Yang group developed a highly sensitive electrochemical sensing platform for the detection of organic dye Sudan I basing on the chemically reduced graphene oxide [20]. The Wang group achieved the simultaneously electrochemical detection of Cd^2+^ and Pb^2+^ based on the graphene film, and the detection limits were evaluated to be as low as 0.02 μg L^−1^ for Cd^2+^ and Pb^2+^ [21]. More recently, graphene has displayed huge application potential in terms of improving the electrochemical property of MXenes [22], [23]. On the one hand, the integration of two-dimensional MXene and graphene could effectively avoid their respective restacking problem, which is beneficial to giving full play to the structural property of each component. On the other hand, graphene owns high electrical conductance and MXene possesses excellent hydrophilic property, the formation of two-dimensional graphene@MXene hybrid could generally exhibit unexpected synergistic effect, which is also conductive to promoting the electrochemical sensing performance. To date, multiple graphene@MXene hybrids were obtained *via* various synthesis strategy, including mechanical mixing method, self-assembly method, hydrothermal method, heat treatment method and reagent reduction treatment method [24], [25], [26], [27], [28]. However, it should be noted that almost all the graphene that used for graphene@MXene hybrids were graphene oxide or reduced graphene oxide that prepared by chemical oxidation/reduction method, which generally involved the complex and dangerous operation procedure, and the highly toxic chemical reagent. With this in mind, how to prepare graphene@MXene hybrid through a simple, safe, low-cost and eco-friendly synthetic route is becoming an urgent need.

In recent years, ultrasound-assisted synthesis method has been considered to be a kind of simple, green, low-cost and promising technology for preparing various functional nanomaterials. During the sonication process, plenty of cavitation bubbles were generated and then collapsed in the liquid environment, the collapsed cavitation bubbles can result in instantaneous high temperature and high pressure, which make it possible to actuate certain physical/chemical reaction and obtain the nanomaterials with different morphology and composition [29]. For example, Ru nanoparticles decorated NiFe layered double hydroxide with excellent hydrogenation catalytic activity [30], cyclodextrin metal–organic framework with superior antibacterial activity [31], platinum-cobalt modified carbon-encapsulated polyaniline with substantial oxygen reduction reaction activity [32] and so on have been successfully obtained through ultrasound-assisted synthesis methods. In addition, according to our previous studies, ultrasound-assisted liquid-phase exfoliation approach could be also used for the preparation of high-quality pristine graphene by breaking the Van der Waals interactions between the layers of bulk graphite. More importantly, the liquid-phase exfoliated graphene suspension generally owned better electrochemical sensing performance for its high graphitization degree, low surface defect and fast electron transfer kinetics compared with the widely used reduced graphene oxide [33], [34], [35], [36], [37]. Taking into account that MXene nanosheets were generally prepared by sonication exfoliation and its abundant surface functional group could make it stable in many solvents, this encourages us to think that whether novel graphene@MXene hybrid could be effectively obtained by simple sonication-assisted synthesis strategy. In this graphene@MXene hybrid, the hydrophilic MXene surface is theoretically conductive to strengthening the interface interaction and promoting the accumulation quantity of the target molecule for the possible hydrogen-bonding or electrostatic adsorption effect, the liquid-phase exfoliation of graphene nanosheets could prevent the aggregation of the MXene nanosheets and facilitate the interface electron transfer. Thus, benefiting from the synergistic effect between MXene nanoflakes and pristine graphene nanosheets, electrochemical sensing platform with excellent performance may be achieved.

In this work, novel graphene@MXene hybrid was prepared through simple, green and eco-friendly ultrasound-assisted liquid-phase exfoliation approach for the first time (Scheme 1). It is found that the liquid-phase exfoliated graphene owned excellent electron transfer property while the hydrophilic MXene possessed outstanding physical adsorption ability toward the target analyte. Benefiting from the synergistic effect between graphene and MXene (Ti_3_C_2_T_x_) nanosheets, the as-synthesized graphene@MXene hybrid exhibited impressive signal enhancement effect toward the oxidation of chlorpromazine and rhodamine B. Chlorpromazine is a kind of common central nervous system drugs, which may be illegally added to the animal feed to reduce the animal activity and promote animal weight. Excessive intake of chlorpromazine may increase the liver damage risk of humans [38]. In addition, rhodamine B is a basic industrial dye and sometimes used as food coloring agent in condiments by some illegal businesses, owning high toxicity, carcinogenicity and mutagenicity [39]. Based on the excellent electrochemical property of the as-synthesized graphene@MXene hybrid, a novel electrochemical sensing platform for the simultaneous voltammetric detection of chlorpromazine and rhodamine B was developed, and the sensing performance can be comparable with many other reported electrode materials (Table S1 and 2, Supplementary Information). Furthermore, the proposed electrochemical method was successfully used to quantify the real content of chlorpromazine in pork sample and rhodamine B in chili powders sample. It is believed that the proposed liquid-phase exfoliation strategy will serve as a typical benchmark for the preparation of graphene@MXene based electrode materials with high electrochemical activity and sensing performance.

**Schehme 1:**
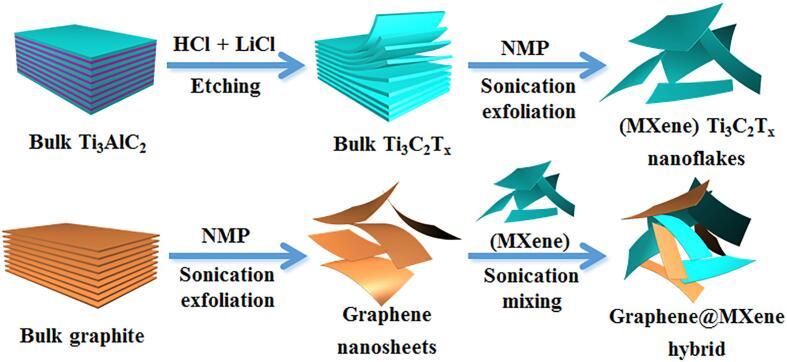
Illustration for the synthesis of liquid-phase exfoliated graphene@MXene hybrid.

## 2 Experimental section

### 2.1 Synthesis of MXene and graphene nanosheets

First, 1.6 g LiF powder was mixed with 20 mL HCl (9 mol/L) until the LiF powder was fully dissolved. After then, 1 g pristine Ti_3_AlC_2_ powder was added and kept stirring for 24 h at 40 °C. Subsequently, the suspension was treated by centrifugation and the obtained solid residue was repeatedly washed by deionized water until the pH of the supernatant reached to approximately 7. The final solid product was then dried in vacuum drying oven at 60 °C for 12 h, and the obtained powder was denoted as etched Ti_3_AlC_2_ powder. After that, 0.5 g etched Ti_3_AlC_2_ powder and 0.5 g graphite powder were respectively added into 25 mL NMP with initial concentration of 20 mg mL^−1^, and sonicated for 5 h in an ultrasonicator (40 kHz, 100 W) under the persistent nitrogen gas protection, and the sonication temperature was controlled at about 30 °C. At last, the obtained graphene and MXene suspensions were centrifuged at 3000 rpm for 60 min to remove the large particles, and the resulted colloidal suspensions consisted of MXene (Ti_3_C_2_T_x_) nanosheets or graphene nanosheets were then separately collected. The concentrations of the MXene and graphene suspensions were then evaluated to be about 0.78 mg mL^−1^ and 0.18 mg mL^−1^ by weighing method, respectively. Besides, MXene and graphene powders were also obtained by filtrating the resulted MXene or graphene suspension. When studying the effect of types of solvents with different viscosity, sonication time and sonication temperature on the structure of graphene and MXene, similar experiment procedures were conducted.

### 2.2 Synthesis of graphene@MXene hybrid

Graphene@MXene hybrid was easily prepared by mixing the liquid-phase exfoliated MXene and graphene suspensions with certain volume ratio. For a typical procedure, 100 μL of MXene suspension (0.78 mg mL^−1^) was mixed with 900 μL of graphene suspension (0.18 mg mL^−1^) and then sonicated for 10 min to obtain the homogeneously dispersed graphene@MXene hybrid suspension. In order to prevent the oxidation of MXene nanoflakes, the sonication mixing process of MXene and graphene nanosheets was also conducted under the persistent nitrogen protection. Basing on this, the volume ratio of MXene in the graphene@MXene suspension was 10 vol% and the total weight concentration of the as-synthesized graphene@MXene hybrid was calculated to be about 0.24 mg mL^−1^. Besides, the weight fractions of MXene and graphene in the hybrid were then calculated to be 32.5 % and 67.5 %, respectively. Other graphene@MXene hybrids with various composition were also prepared *via* changing the volume ratio of MXene suspension to 2.5 vol%, 5.0 vol%, 20 vol% and 30 vol%, the weight fractions of MXene in these hybrids were then assessed to be about 10.0 %, 18.6 %, 52.0 % and 65.0 %, respectively.

### 2.3 Preparation of real samples

The real pork and red chilli powder samples were purchased from the local food market. Before the detection of chlorpromazine in pork and rhodamine B in red chilli powder, the samples were treated by the following procedures [40], [41]. 5 g smashed pork was first mixed with 15 mL acetonitrile and sonicated for 15 min. After then, another 15 mL acetonitrile was added into the pork sample to extract chlorpromazine again. The extraction solution was then collected by centrifuging at 5000 rpm for 10 min at room temperature. 15 mL *n*-hexane was then added into the extraction solution and vigorously shaken for 10 min. The mixture was then centrifuged at 5000 rpm for 10 min again. The subnatant was collected and evaporated to dryness at 45 °C with low pressure. After that, the residue was redissolved in 5 mL methanol, and the solution was filtered and collected through a 0.22 μm filter membrane. Finally, 2 mL filter liquor was added to 2 mL phosphate buffer solution (0.1 M, pH = 6.5) for further electrochemical detection. For the detection of rhodamine B in red chilli powder, 1 g red chilli powder was first dispersed into 10 mL acetonitrile and sonicated for 20 min. The mixture was then centrifuged at 5000 rpm for 10 min. The supernatant liquid was collected and another 10 mL acetonitrile was further used to extract rhodamine B from the residual solid again. When the extract solution was mixed together, 1 g NaCl and 2 mL water was added in succession, and the mixed solution was placed in a refrigerator with −20 °C for 5 h to remove the natural pigments and lipids. After that, the resulted solution was centrifuged at 5000 rpm for 10 min, and the upper acetonitrile solution was collected for the following electrochemical analysis. Spiked samples were obtained by adding a certain amount of chlorpromazine or rhodamine B standard before the pretreatment.

## 3 Results and discussion

### 3.1 Structure analysis of graphene@MXene hybrid

**Fig. 1:**
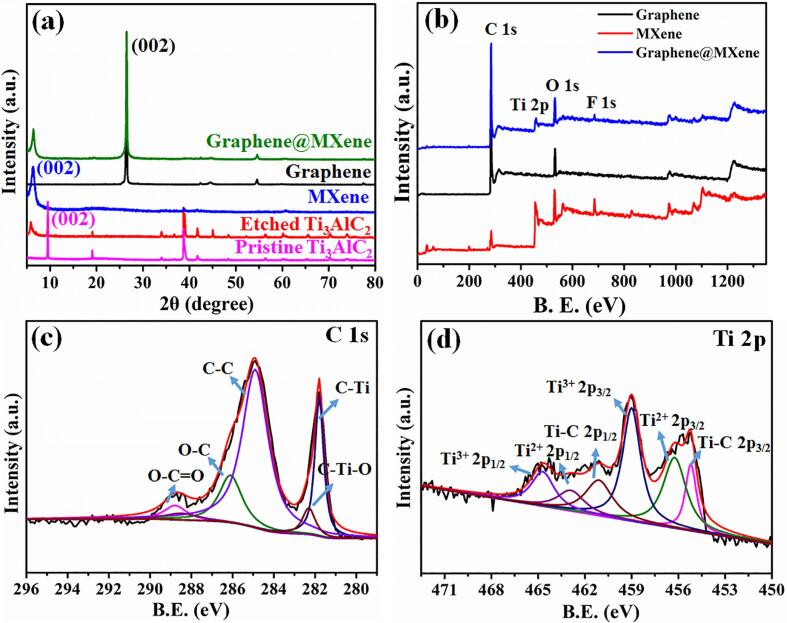
(a) XRD patterns of the pristine Ti_3_AlC_2_, HF-etched Ti_3_AlC_2_, ultrasound-assisted synthesis of graphene, MXene (Ti_3_C_2_T_x_) and graphene@MXene powders. (b) XPS survey spectrum, high-resolution (c) C 1 s and (d) Ti 2p spectra of graphene@MXene hybrid.

XRD patterns of the pristine Ti_3_AlC_2_, HF-etched Ti_3_AlC_2_, ultrasound-assisted synthesis of graphene, MXene (Ti_3_C_2_T_x_) and graphene@MXene powders were first obtained to study their crystal structure (Fig. 1a). It is seen that after acid etching treatment, a new diffraction peak located at 2θ = 6.2° was appeared compared with the pristine Ti_3_AlC_2_ powders, which could be assigned to the (0 0 2) crystal face of the layer-like Ti_3_C_2_T_x_ material. This result suggested that only partial Al element was removed from the pristine MAX phase, and the HF-etched Ti_3_AlC_2_ powders were composed of bulk Ti_3_AlC_2_ and Ti_3_C_2_T_x_. Compared with the pristine bulk Ti_3_AlC_2_ and HF-etched Ti_3_AlC_2_, their characteristic peaks that located at about 2θ = 39° disappeared in MXene, demonstrating the Al element was successfully removed and only the bulk Ti_3_C_2_T_x_ powders in the HF-etched Ti_3_AlC_2_ can be exfoliated into thin-layer of Ti_3_C_2_T_x_ nanosheets. Besides, the (0 0 2) diffraction peak of MXene shifted to a lower 2θ angle value relative to that of bulk Ti_3_AlC_2_, demonstrating the enlarged interlayer spacing, which could be also attributed to the effective etching of the Al layer [22]. Besides, a well-defined peak at 2θ = 26.0° was observed for the liquid-phase exfoliated graphene, which can be indexed to the characteristic (0 0 2) crystal face of carbon materials, suggesting the resulted graphene nanosheets owned high graphitization degree. According to the characteristic diffraction peaks of graphene@MXene powders at 6.2° and 26.0°, it is deduced that graphene@MXene hybrid was successfully prepared through the simple ultrasound-assisted synthesis method.

X-ray photoelectron spectroscopy (XPS) analysis was further conducted to study the surface chemical states and composition of the as-synthesized graphene nanosheets, MXene nanosheets, and graphene@MXene hybrid (Fig. 1b). The XPS survey spectrums suggested that the as-synthesized MXene nanosheets was mainly composed of Ti, C, O and F elements while the liquid-phase exfoliated graphene was made up of C and O element. It is generally believed that the O and F elements of MXene nanosheets was resulted by the HF etching process [12]. Besides, it is seen that the element composition of graphene@MXene hybrid was similar to that of pure MXene nanosheets. However, the signal intensity of C element in graphene@MXene hybrid was obviously strengthened compared with pure MXene, which could be ascribed to the introduction of graphene nanosheets, further demonstrating the formation of graphene@MXene hybrid. In addition, the signal of Al element was not detected in MXene and graphene@MXene materials, suggesting only the multi-layered Ti_3_C_2_ precursors can be exfoliated into the flake-like nanostructure. High-resolution XPS spectra of the C 1 s was divided into five well-defined peaks that located at 281.8, 282.3, 284.8, 286.2 and 288.8 eV, which could be ascribed to C-Ti, C-Ti-O, C—C, C—O and O=C-O bonds, respectively (Fig. 1c). In addition, Ti 2p of graphene@MXene hybrid was fitted into three pairs of peaks (Ti 2p_3/2_/Ti 2p_1/2_), which could be assigned to the Ti-C (455.2 eV/461.1 eV), Ti^2+^ (456.2 eV/462.9 eV), and Ti^3+^ (458.9 eV/464.7 eV) bonds (Fig. 1d) [22], [23], [24]. According to the XRD and XPS analysis, it is deduced that graphene@MXene hybrid was successfully synthesized.

**Fig. 2:**
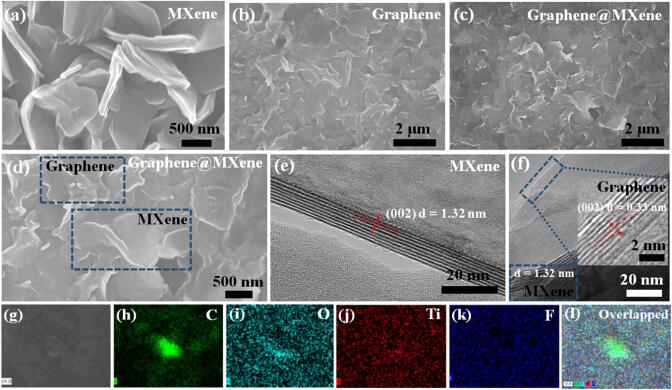
SEM images of (a) MXene nanosheet, (b) graphene nanosheet and (c, d) graphene@MXene hybrid with different magnification. High-resolution TEM images of (e) MXene nanosheets and (f) graphene@MXene hybrid. (g-l) Element mapping analysis of graphene@MXene hybrid.

The surface morphology and structure of different materials were then characterized by SEM and TEM. Figure S1 (Supplementary Information) displayed the morphology of the pristine graphite and Ti_3_AlC_2_ powders, both graphite and Ti_3_AlC_2_ displayed irregular shape with size ranging from 5 to 30 μm. After etching by HF, only partial particles exhibited the accordion-like morphology (Figure S2, Supplementary Information), suggesting the Al element was incompletely removed from the precursor, and the etched Ti_3_AlC_2_ was composed of multi-layers Ti_3_C_2_T_x_ and raw Ti_3_AlC_2_ particles, which was consistent with the XRD analysis. When the etched Ti_3_AlC_2_ particles and graphite powders were treated by sonicating for 5 h in *N*-methyl-2-pyrrolidone (NMP), numerous nanoflakes were observed for the MXene (Fig. 2a) and graphene (Fig. 2b) with lateral size ranging from 500 nm to 1.5 μm, suggesting the accordion-like Ti_3_C_2_T_x_ particles and graphite precursors can be effectively transformed into the laminar nanomaterial by simple liquid-phase exfoliation strategy. As to the graphene@MXene hybrid, layered stacking structure that composed of two-dimensional MXene and graphene with uniform distribution was observed (Fig. 2c and d). It is no doubt that the mutually intertwined structure can effectively avoid the re-stacking problem of graphene and MXene. In addition, according to the high-resolution TEM images of MXene nanosheets (Fig. 2e) and graphene@MXene (Fig. 2f), we could further speculate the formation of the two-dimensional hybrid due to the remarkably different interlayer spacing of the (0 0 2) crystal plane of MXene and graphene [23]. Besides, the effective integration of graphene and MXene nanosheets can also be observed from the low-magnification TEM image of graphene@MXene hybrid (Figure S3, Supplementary Information). Energy dispersive X-ray (EDX) elements mapping analysis suggested that the obtained graphene@MXene hybrid was consisted of Ti, C, O and F elements, which was in agreement with the XPS analysis. The distribution of partial carbon element was highly overlapped with that of Ti, O and F elements, indicating the existence of the hydrophilic Ti_3_C_2_T_x_. Besides, partial carbon element signal was notably stronger than the adjacent Ti, O and F elements, suggesting the existence of graphene nanosheets (Fig. 2g-l).

**Fig. 3:**
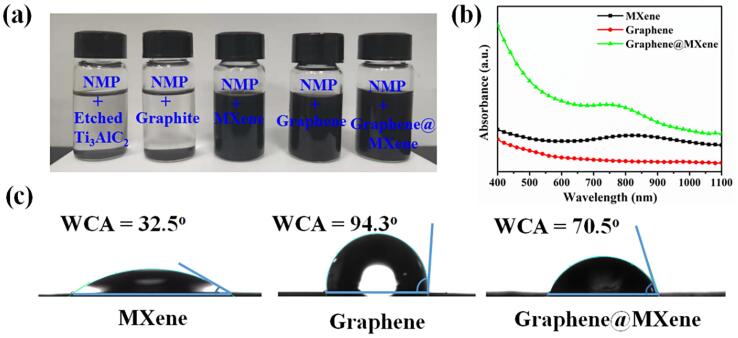
(a) Optical images of etched Ti_3_AlC_2_ and pristine graphite powders in NMP solvent before and after sonication treatment. (b) UV–vis adsorption spectrum of MXene, graphene and graphene@MXene hybrid suspensions. (c) Water contact angles of different electrode interface.

In order to further highlight the feasibility of the proposed ultrasound-assisted liquid-phase exfoliation strategy for synthesizing graphene@MXene hybrid, the optical images of etched Ti_3_AlC_2_ and pristine graphite in NMP before and after sonication treatment were provided (Fig. 3a). It is seen that both the etched Ti_3_AlC_2_ and pristine graphite powders were deposited on the bottom of the glass bottles without sonication treatment. After sonication exfoliation and centrifugal screening treatment, stable MXene and graphene nanosheets suspensions were obtained, suggesting ultrasound-assisted liquid-phase exfoliation technology can be used to prepare nano-size material from its bulk precursors. Basing on this, stable graphene@MXene hybrid were also achieved in NMP solvent by simple sonication mixing operation. After then, the UV–vis adsorption spectrum of MXene, graphene and graphene@MXene hybrid suspensions were obtained (Fig. 3b). There was no obvious characteristic absorption peak for the graphene suspension, while MXene and MXene@graphene exhibited similar optical adsorption feature with maximum absorption wavelength at about 800 nm, which was consistent with the reported result [42], suggesting the existence of MXene nanosheets. Furthermore, water contact angle (WCA) test was conducted to evaluate the surface property of different materials (Fig. 3c). The WCA value of MXene was only 32.5°, displaying obvious hydrophilic property. By contrast, the resulted liquid-phase exfoliated graphene was hydrophobic (WCA = 94.3°). Due to the introduction of MXene nanosheets, the WCA value of graphene decreased from 94.3° to 70.5°, indicating the as-synthesized graphene@MXene hybrid was hydrophilic, which may be conducive to promoting the interaction between the graphene-based electrode interface and the polar analytes that resulted by the hydrogen-bonding or electrostatic adsorption effect.

### 3.2 Effect of sonication parameters on the structure and electrochemical property of graphene@MXene hybrid

**Fig. 4:**
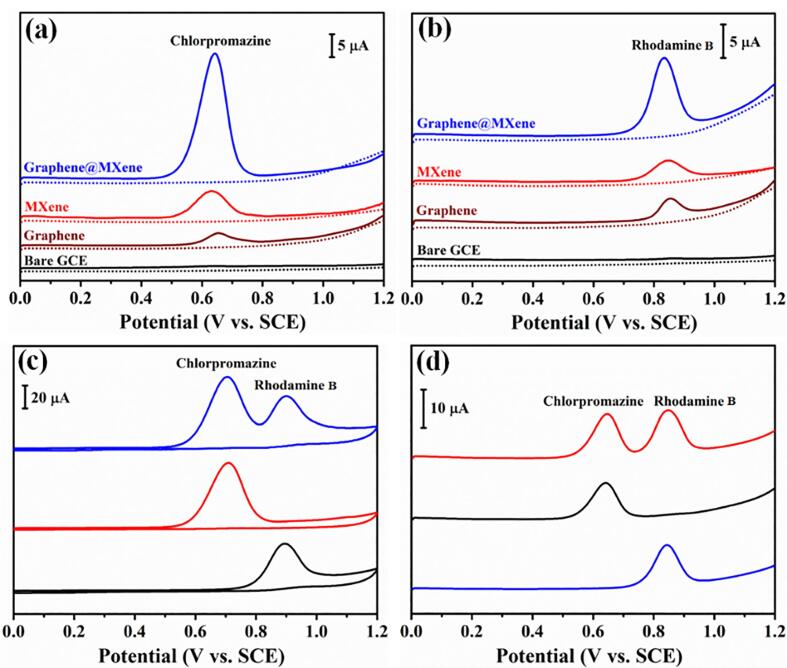
DPV curves of bare GCE, MXene, graphene and graphene@MXene hybrid in the absence and presence of (a) 1 μM chlorpromazine and (b) 1 μM rhodamine B. (c) CV curves of graphene@MXene hybrid for 10 μM chlorpromazine, 50 μM rhodamine B, and the coexistence of 10 μM chlorpromazine and 50 μM rhodamine B with scan rates of 100 mV^−1^. (d) DPV curves of graphene@MXene hybrid for 0.25 μM chlorpromazine, 1 μM rhodamine B, and the coexistence of 0.25 μM chlorpromazine and 1 μM rhodamine B. Accumulation potential: 0 V, amount of suspension: 3 μL, accumulation time: 120 s.

The electrochemical property of graphene@MXene hybrid was first study in 0.1 M, pH 6.5 phosphate buffer solution by differential pulse voltammetry (DPV) scanning in the absence and presence of 1 μM chlorpromazine (Fig. 4a) and 1 μM rhodamine B (Fig. 4b). It is seen that there was no any oxidation peak in the blank electrolyte for bare GCE, MXene, graphene and graphene@MXene hybrid. Compared with bare GCE, a sharp peak that corresponded to the oxidation of chlorpromazine (0.65 V) and rhodamine B (0.85 V) appeared at MXene and graphene, demonstrating the liquid-phase exfoliated MXene and graphene are highly active toward the oxidation of chlorpromazine and rhodamine B. Surprisingly, the oxidation peak signals of chlorpromazine and rhodamine B were greatly enhanced at graphene@MXene hybrid, exhibiting excellent synergetic enhancement effect. Fig. 4c illustrated the cyclic voltammetry (CV) curves of graphene@MXene hybrid for 10 μM chlorpromazine, 50 μM rhodamine B, and the coexistence of 10 μM chlorpromazine, 50 μM rhodamine B. Clearly, the oxidation of chlorpromazine and rhodamine B at graphene@MXene was irreversible. Besides, the resulted response currents in the mixed solution of chlorpromazine and rhodamine B were closed to that of separate chlorpromazine or rhodamine B, respectively, suggesting the synthesized graphene@MXene hybrid was qualified for the simultaneous electrochemical analysis of chlorpromazine and rhodamine B. Similar phenomenon was also observed for the DPV test, there was no mutual effect for their respective oxidation (Fig. 4d). In order to achieve stronger sensing performance, graphene@MXene hybrids with different composition were also obtained by changing the volume ratio of MXene suspension (Figure S4, Supplementary Information). When the volume ratio of MXene suspension was 10 vol%, the largest current responses were obtained for the oxidation of chlorpromazine and rhodamine B. This means when the weight fractions of graphene and MXene were 67.5 % and 32.5 %, respectively, the optimal synergistic effect was achieved. Thus, graphene@MXene with 67.5 wt% of graphene was selected as the final electrode material for the further electrochemical test.

Besides, in order to investigate the optimal ultrasound-assisted synthesis condition, the effect of sonication parameters, including types of solvents with different viscosity, sonication time and sonication temperature on the structure and electrochemical sensing performance of graphene@MXene hybrid has been systematacially studied. Figure S5 (Supplementary Information) illustrated the influence of solvents types on the exfoliation efficiency and stability of graphene and MXene. The selected solvents contained methanol (MeOH), DMF, H_2_O, ethanol (EtOH), NMP, DMSO, and their viscosity coefficient were 0.553, 0.802, 0.894, 1.07, 1.65, 1.99 mPa.s, respectively. It is seen that bulk graphite powder can be exfoliated into graphene nanosheets in DMF and NMP while bulk etched Ti_3_AlC_2_ can be exfoliated into MXene nanoflakes in H_2_O and NMP. Considering only NMP can be used for the synchronous exfoliation of graphene and MXene, thus NMP was selected as the optimum solvent to prepare graphene, MXene (Ti_3_C_2_T_x_) and graphene@MXene nanoflakes suspensions. More importantly, both the resulted graphene and MXene nanosheets exhibited good stability in NMP. Studies showed that only when the surface energy of solvent was closed to that of the target material, bulk two-dimensional precursors can be easily transformed into the sheet-like nanomaterial through sonication-assisted liquid-phase exfoliation [43], [44], it is no doubt that NMP was a kind of ideal exfoliation solvent for the preparation of graphene and MXene nanosheets. Figure S6 (Supplementary Information) displayed the optical images of graphene and MXene suspensions that prepared in NMP with different sonication time. It is seen that graphene and MXene nanoflakes can be easily obtained only after 1 h of ultrasound treatment. In addition, the concentration of graphene suspension gradually increased with the increase of sonication time. In comparison, the concentration of MXene suspension changed little when the sonication time exceeded 5 h (Figure S7a and b, Supplementary Information). Thus, the longer the sonication time, the higher the concentration of graphene@MXene hybrid suspension (Figure S7c, Supplementary Information). Figure S7d (Supplementary Information) showed the weight fraction variation of graphene and MXene in the graphene@MXene hybrid suspension with sonication time. The weight fraction of MXene gradually increased when the sonication time was ranging from 1 to 5 h. Further increasing the sonication time, the weight fraction of MXene decreased, which can be ascribed to the remarkably increased graphene concentration. Besides, the effect of sonication time on the surface morphology of graphene and MXene was studied (Figure S8, Supplementary Information). It is seen that the morphology of graphene and MXene changed little when the sonication time was fixed at 1 h, 5 h or 9 h. Furthermore, different sonication time of graphene@MXene hybrid suspensions were prepared and their DPV curves were collected in the presence of 1 μM chlorpromazine and 1 μM rhodamine B in 0.1 M, pH 6.5 of phosphate buffer solution (Figure S9, Supplementary Information). When the sonication time increased from 0 to 5 h, the oxidation peak current of chlorpromazine and rhodamine B gradually strengthened. This may be ascribed to the increased loading amount of the active graphene and MXene nanoflakes on the electrode surface. Further increasing the sonication time to 9 h, the oxidation peak current of chlorpromazine and rhodamine displayed gradual decline. This phenomenon may be caused by the greatly increased graphene weight ratio in the graphene@MXene hybrid, which remarkably affected the synergistic effect between graphene and MXene nanosheets. Thus, the sonication time was fixed at 5 h. After then, the effect of sonication temperature on the concentration, morphology of graphene and MXene suspensions, and the electrochemical sensing performance of graphene@MXene hybrid was studied. During the sonication process, the NMP solvent was saturated by persistent N_2_ flow. It is seen that when the sonication temperature varied from 10 °C to 40 °C, the exfoliation efficiency and morphology of graphene and MXene changed little (Figure S10 and 11, Supplementary Information). Besides, sonication time has no obvious effect on the electrochemical activity of graphene@MXene hybrid (Figure S12, Supplementary Information). Considering the room temperature was closed to 30 °C, thus 30 °C was selected as the final sonication temperature for convenience.

**Fig. 5:**
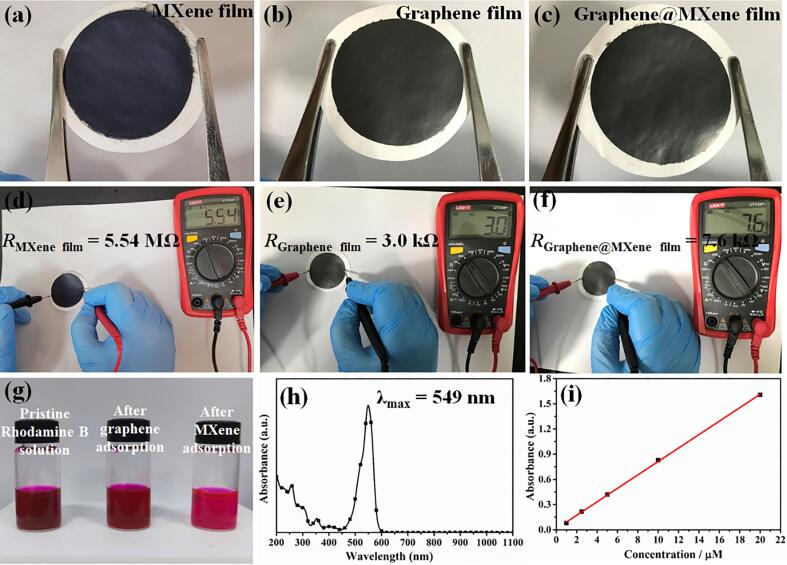
Optical images and resistance for the flexible (a, d) MXene, (b, e) graphene and (c, f) graphene@MXene hybrid films. (g) Photographs of rhodamine B solutions before and after MXene or graphene adsorption treatment. (h) UV–vis adsorption spectrum of rhodamine B solution. (i) Linear plots between the absorbance and rhodamine B concentrations.

Undoubtedly, the as-synthesized graphene@MXene hybrid displayed excellent electrochemical sensing property. For probing the origin of its superior electrochemical activity, the electron transfer ability and adsorption property of different materials were studied. MXene, graphene and graphene@MXene suspensions with same concentration (0.15 mg mL^−1^) and volume (20 mL) were first treated by vacuum filtration on the organic porous films, we could see that compact and flat flexible films were easily obtained (Fig. 5a-c), this may be due to their two-dimensional flake-like structure. After then, the resistance (*R*) values of MXene, graphene, and graphene@MXene hybrid were determined as 5.54 MΩ, 3.0 kΩ and 7.6 kΩ, respectively (Fig. 5d-f). It is no doubt that the conductivity of graphene is far better than that of MXene, and the introduction of graphene greatly improved the electron transfer ability of MXene. Electrochemical impedance spectrum (EIS) tests for different electrode materials were then carried out at applied potential of 0.9 V in 0.1 M, pH = 6.5 of phosphate buffer solution containing 0.1 M chlorpromazine solution (Figure S13, Supplementary Information). In the EIS Nyquist plots, the wider the semicircle diameter, the lager the charge transfer resistance. Compared with pure MXene, the interfacial electron transfer resistance was substantially decreased at graphene@MXene hybrid, which can be attributed to the excellent electron transfer ability of the ultrasound-assisted synthesis of graphene material. After then, the physical adsorption property of different material were evaluated by determining the absorbance of rhodamine B solution before and after MXene or graphene adsorption. Specifically, 10 mg MXene or graphene powders were uniformly dispersed in 5 mL, 1 mM rhodamine B solution and continually stirred for 24 h, the supernatant liquors were then collected by high-speed centrifugation (Fig. 5g). Compared with the pristine rhodamine B solution, a slightly faded rhodamine B solution was observed after graphene adsorption treatment. Surprisingly, the rose colour of rhodamine B has been changed to pink colour after MXene adsorption treatment, suggesting more rhodamine B molecules have been removed by MXene, and the adsorption ability of MXene was far better than that of graphene. This may be benefited from its abundant surface functional group (OH, O, and/or F), which could provide more molecular action sites toward the target analyte. In order to evaluate the adsorption ability of graphene and MXene more accurately, the absorbance of rhodamine B solution after MXene or graphene adsorption was determined at the maximum absorption wavelength (Fig. 5h). Basing on the linear plots between the absorbance and rhodamine B concentrations (Fig. 5h), the concentrations of rhodamine B solution were calculated as 0.372 mM after MXene adsorption and 0.95 mM after graphene adsorption. Thus, the excellent electrochemical sensing performance of graphene@MXene hybrid can be attributed to the synergistic effect between the liquid-phase exfoliated graphene and MXene nanosheets. In this hybrid, graphene could greatly facilitate the interfacial electron transfer rate while the hydrophilic MXene could remarkably promote the adsorption quantum of the target analytes.

### 3.3 Electrochemical detection of chlorpromazine and rhodamine B

**Fig. 6:**
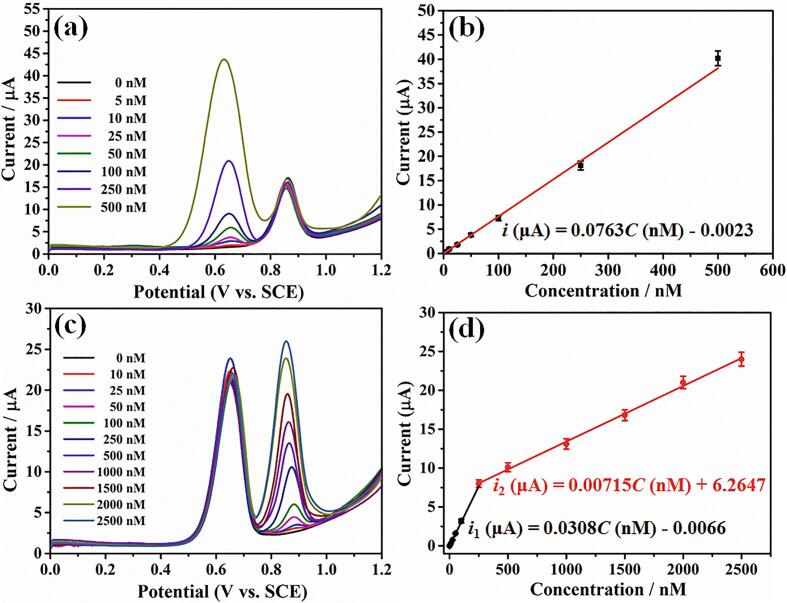
(a) DPV curves of different concentrations of chlorpromazine in the presence of 1 μM rhodamine B. (b) Linear plots of the oxidation peak currents *versus* concentrations for chlorpromazine. (c) DPV curves of different concentrations of rhodamine B in the presence of 0.25 μM chlorpromazine. (d) Linear plots of the oxidation peak currents *versus* concentrations for rhodamine B.

In order to achieve the highly sensitive detection of chlorpromazine and rhodamine B, the effect of pH of electrolytes, accumulation potential, accumulation time and amount of suspension on the current responses of chlorpromazine and rhodamine B at the surface of graphene@MXene hybrid were investigated (Figure S14, Supplementary Information). It is seen that the optimal electrolyte was pH 6.5 of phosphate buffer solution (0.1 M), accumulation potential was −0.1 V, accumulation time was 150 s and amount of suspension was 5 μL. Under the optimal conditions, the linear detection range and detection limit of chlorpromazine and rhodamine B at graphene@MXene hybrid was studied by DPV. Fig. 6a displayed the DPV curves of different concentrations of chlorpromazine in the presence of 1 μM rhodamine B, while Fig. 6c showed the DPV curves of different concentrations of rhodamine B in the presence of 0.25 μM chlorpromazine. The oxidation peak currents (*i*, μA) of chlorpromazine and rhodamine B linearly raised with their continuously increased concentrations (*C*, nM), and the corresponded linear relationships can be depicted as: *i* = 0.0763*C* − 0.0023 for chlorpromazine in the range of 5 nM to 500 nM, and *i*_1_ = 0.0308*C* − 0.0066 in the range of 10 nM to 250 nM and *i*_2_ = 0.00715*C* + 6.2647 in the range of 250 nM to 2500 nM for rhodamine B. Based on the 3σ/m criterion, where σ is the standard deviation of ten DPV tests for the blank electrolyte and m is the slope of the linear plots, the detection limits of chlorpromazine and rhodamine B were then evaluated to be as low as 1.25 nM and 2.45 nM, respectively.

The reproducibility of graphene@MXene hybrid modified electrodes was then evaluated by recording the repetitive DPV curves and current responses of 0.25 μM chlorpromazine and 1 μM Rhodamine B at ten different electrodes. The relative standard deviation (RSD) was then measured to be about 3.8 % for chlorpromazine and 4.8 % for rhodamine B, confirming its good reproducibility.

Stability evaluation was conducted by recording the oxidation peak currents of 0.25 μM chlorpromazine and 1 μM rhodamine B at the same sensor for every day. The fabricated sensor was used for only once for each test. After each electrochemical test, the GCE was polished with 0.05 μm alumina powder, sonicated in deionized water and ethanol, modified with 5 μL graphene@MXene suspension, and drying under an infrared lamp in the air. After that, the graphene@MXene modified GCE was stored in air at room temperature for different time for the next test. It is found that the current responses of chlorpromazine and rhodamine B at graphene@MXene hybrid modified electrode still can reached to 93.1 % and 91.2 % of their initial values after two weeks, respectively, demonstrating the long-term stability of graphene@MXene hybrid modified electrodes. For further judging the stability of graphene@MXene hybrid modified electrode in the electrolyte solution, graphene@MXene modified GCE was immersed in the blank electrolyte solution for different time and the oxidation peak signals of 0.25 μM chlorpromazine and 1 μM rhodamine B at graphene@MXene modified GCE was then recorded by DPV test (Figure S15, Supplementary Information). It is found that when the graphene@MXene modified GCE was immersed in the buffer solution for 60 min, both the electrochemical oxidation signals of chlorpromazine and rhodamine B could still reached to about 90 % of its original values. Further extending the immersing time to 90 min, relatively obvious current drops were appeared, which may be because the loaded graphene@MXene material was peeled off from the GCE surface. This result manifested the graphene@MXene modified GCE still owned satisfied stability in the electrolyte solution.

Anti-interference ability of graphene@MXene hybrid modified electrodes was also investigated. The potential interference substances were severally adding into 0.25 μM chlorpromazine or 1 μM rhodamine B and the oxidation peak currents of chlorpromazine and rhodamine B were then recorded. It was found that 500 folds of Na^+^, K^+^, Mg^2+^, Ca^2+^, Fe^3+^, Cu^2+^, SO_4_^2−^,Cl^−^, NO_3_^−^, 200 folds of moxifloxacin metronidazole, ofloxacin, fleroxacin, erythrocin, gatifloxacin, dinotefuran has no obvious effect on the electrochemical response of 0.25 μM chlorpromazine. 500 folds of Na^+^, K^+^, Mg^2+^, Ca^2+^, Fe^3+^, Cu^2+^, SO_4_^2−^,Cl^−^, NO_3_^−^, 100 folds of ascorbic acid, congo red, quinoline yellow, lemon yellow, alizarin red, carmine has no obvious effect on the electrochemical response of 1 μM rhodamine B. These results manifested the as-synthesized graphene@MXene hybrid owned strong anti-interference ability for the detection of chlorpromazine and rhodamine B.

In order to further verify the feasibility of the electrochemical sensor, the content of chlorpromazine in pork sample and rhodamine B in red chilli powder sample were evaluated under the optimal test conditions by DPV. Each sample was parallelly detected for three times, and the content of the target analyte was measured by standard addition method. The analysis results were summarized in Table 1, the recovery ratio was ranging from 93.50 % to 97.60 % with RSD values lower than 5 %, suggesting good accuracy and application potential of the new electrochemical method in real samples analysis.

**Table 1:** Practical samples analysis of the electrochemical method.

Samples	Analyte	Detected (μM)	Added (μM)	Found (μM)	Recovery (%)	RSD (%)
Pork	chlorpromazine	–	0.2	0.187	93.50	3.97
			0.5	0.471	94.20	3.54
Chilli powder	rhodamine B	0.055	0.05	0.0476	95.20	3.29
			0.1	0.0976	97.60	2.98

## 4 Conclusion

Ultrasound-assisted liquid-phase exfoliation method has been confirmed to be a simple and effective method for the preparation of novel graphene@MXene hybrid. Due to the synergistic effect, the as-synthesized two-dimensional graphene@MXene hybrid was endowed with superior electron transfer ability and physical adsorption property. Basing on this, excellent electrochemical activity toward the oxidation of chlorpromazine and rhodamine B was achieved, and a facile and sensitive electrochemical sensor was established for the simultaneous voltammetric detection of chlorpromazine and rhodamine B. Besides, the fabricated sensor displayed satisfied reproducibility, stability and practicability. Considering the diversity of MAX phase materials, the proposed liquid-phase exfoliation strategy is believed to be instructive for preparing other graphene@MXene hybrids for various applications.

## CRediT authorship contribution statement

**Shenchao Shi:** Methodology, Conceptualization, Software, Visualization, Validation, Writing – original draft, Writing – review & editing. **Ruizheng Zhong:** Methodology, Conceptualization, Formal analysis, Writing – original draft. **Lele Li:** Methodology, Conceptualization. **Chidan Wan:** Supervision, Formal analysis, Writing – review & editing, Funding acquisition, Project administration. **Can Wu:** Supervision, Formal analysis, Writing – review & editing, Funding acquisition, Project administration.

## Declaration of Competing Interest

The authors declare that they have no known competing financial interests or personal relationships that could have appeared to influence the work reported in this paper.

## Data Availability

Data will be made available on request.
