# Supplementary material for: Ultrasound-assisted synthesis of graphene@MXene hybrid: A novel and promising material for electrochemical sensing
Source: Ultrason Sonochem. 2022 Oct 22;90:106208. doi: 10.1016/j.ultsonch.2022.106208 (PMC9626737; doi:10.1016/j.ultsonch.2022.106208)
Supplement: Supplementary data 1 [file mmc1.docx]

**Supplementary Information**

**Ultrasound-assisted synthesis of graphene@MXene hybrid: a novel and promising material for electrochemical sensing**

Shenchao Shi^†#^, Ruizheng Zhong^‡#^, Lele Li^‡^, Chidan Wan^†*^, Can Wu^‡*^

^†^ Department of Hepatobiliary Surgery, Union Hospital, Tongji Medical College, Huazhong University of Science and Technology, Wuhan 430022, China

^‡^ Collaborative Innovation Center for Advanced Organic Chemical Materials Co-constructed by the Province and Ministry, Ministry of Education Key Laboratory for the Synthesis and Application of Organic Functional Molecules, School of Materials Science & Engineering, Hubei University, Wuhan 430062, China

^#^ These authors contributed equally to this work.

* Corresponding author: chidanwanjsr@163.com (C. Wan), [cwu@hubu.edu.cn](mailto:cwu@hubu.edu.cn) (C. Wu).

**Reagents**

Graphite, Ti_3_AlC_2_, lithium fluoride (LiF), hydrochloric acid (HCl, 37wt. %), *N*-methylpyrrolidone (NMP), methanol (MeOH), *N, N*-dimethylacetamide (DMF), ethanol (EtOH), dimethyl sulphoxide (DMSO), chlorpromazine and rhodamine B were purchased from Shanghai Macklin Biochemical Technology Co., Ltd. All the reagents were of analytical grade and used without further purification. The used deionized water throughout the experiment was acquired through a Millipore system.

**Material characterization**

Morphology and structure analysis was conducted on MAGELLAN 400 scanning electron microscope (SEM) and transmission electron microscope (TEM) (JEOL JEM-2100, Japan). X-ray diffraction (XRD) patterns were obtained on X-ray diffractometer (RIGAKU, D/MAX 2550 V) with Cu Ka radiation (λ = 1.5418 Å). X-ray photoelectron spectra (XPS) analysis was studied by AXIS-ULTRA DLD-600W spectrometer (Shimadzu, Japan). Water contact angle (WCA) was measured using the Harke-SPCAcontact angle tester (BEIJIN HAKE, China).

**Electrochemical measurements**

All the electrochemical experiments were conducted by a three-electrode system on a CHI 660E electrochemical workstation, in which graphene@MXene hybrid modified glassy carbon electrode (GCE, diameter 3 mm), platinum wire and saturated calomel electrode (SCE) were used as the working electrode, count electrode and reference electrode, respectively. For the working electrode, 0.1 mL MXene suspension was first mixed with 0.9 mL graphene suspension, 5 μL graphene@MXene hybrid suspension was then dropped on the clean GCE surface until the solvent was completely evaporated. Before the electrochemical test, the working electrode was scaned from 0 to 1.2 V for three cycles in 0.1 M, pH 6.5 phosphate buffer solution to obtain a stable electrode interface. The differential pulse voltammetry (DPV) curves were recorded from 0 to 1.2 V with 50 mV of pulse amplitude, 40 ms of pulse width and 40 mV/s of scan rate. Electrochemical impedance spectrum (EIS) test was carried out at applied potential of 0.9 V with the frequency ranging from 100 kHz to 0.1 Hz in 0.1 M pH 6.0 phosphate buffer solution containing 0.1 M chlorpromazine.

**Table S1.** Recently reported electrochemical sensors for chlorpromazine.

| **Electrodes** | **Linear range**  **(μM)** | **Detection**  **limit (nM)** | **Sensitivity**  **(µA μM^-1^ cm^-2^)** | **Reference** |
| --- | --- | --- | --- | --- |
| Pencil leads | 0.01-0.08 | 3 | 21.94 | [S1] |
| MWCNT-PEI | 0.019-9.2 | 10 | 1.3 | [S2] |
| P3MT/γ-CD | 0.6-10 | 100 | 87.25 | [S3] |
| Graphene paste | 0.01-9 | 6 | 31.14 | [S4] |
| Boron-doped diamond | 0.1-45 | 30 | 0.35 | [S5] |
| GO-Fe nanoparticle/ZnO | 0.02-1047.74 | 20 | 7.56 | [S6] |
| CoNPs | 0.002-1 | 0.6 | 695.2 | [S7] |
| **Graphene@MXene** | **0.005-0.5** | **1.25** | **1090** | **This work** |

MWCNT-PEI: Multiwalled carbon nanotube-polyethyleneimine; P3MT/γ-CD: poly-3-methylthiophene-γ-cyclodextrin; GO: graphene oxide

**Table S2.** Recently reported electrochemical sensors for rhodamine B.

| **Electrodes** | **Linear range**  **(μM)** | **Detection limit (nM)** | **Sensitivity**  **(µA μM^-1^ cm^-2^)** | **Reference** |
| --- | --- | --- | --- | --- |
| Graphene/Ag | 2-100 | 1940 | - | [S8] |
| Cu_2_O-ERGO | 0.01-20 | 6 | 19.29 | [S9] |
| Cu@carbon sphere | 0.3-30 | 100 | - | [S10] |
| MWCNTs-PEI | 0.01-10 | 6 | 47.74 | [S11] |
| Carbon nanotubes paste | 0.1-15 | 200 | 3.315 | [S12] |
| Nd-MOF | 0.08-2.0  2.0-40 | 3.6 | - | [S13] |
| MnO_2_/ERGO | 0.02-1  1-20 | 6 | 72.14  22.04 | [S14] |
| **Graphene@MXene** | **0.01-0.25** | **2.45** | **440** | **This work** |
|  | **0.25-2.5** |  | **102.14** |  |

ERGO: electrochemical reduced graphene oxide; MWCNTs-PEI: polyethylenimine and multi-walled carbon nanotubes composite;


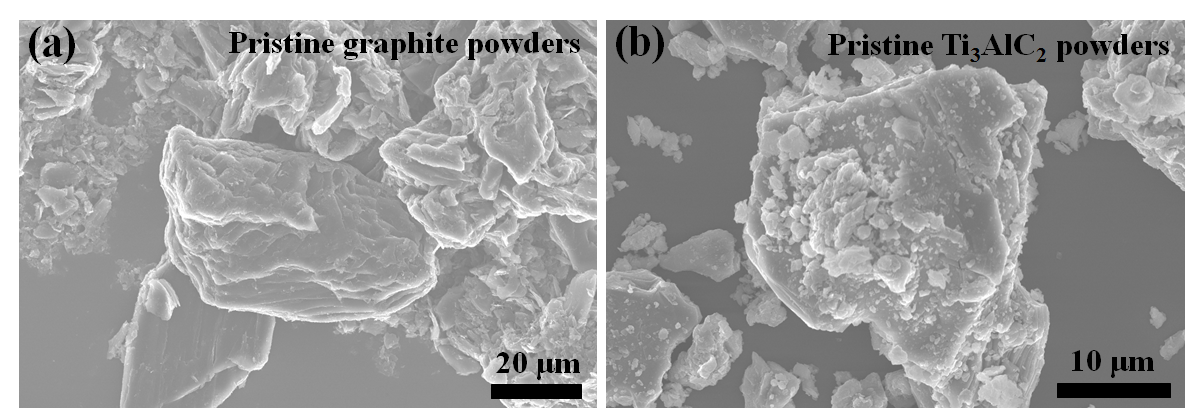


**Figure S1.** SEM images of pristine graphite and Ti_3_AlC_2_ powders.


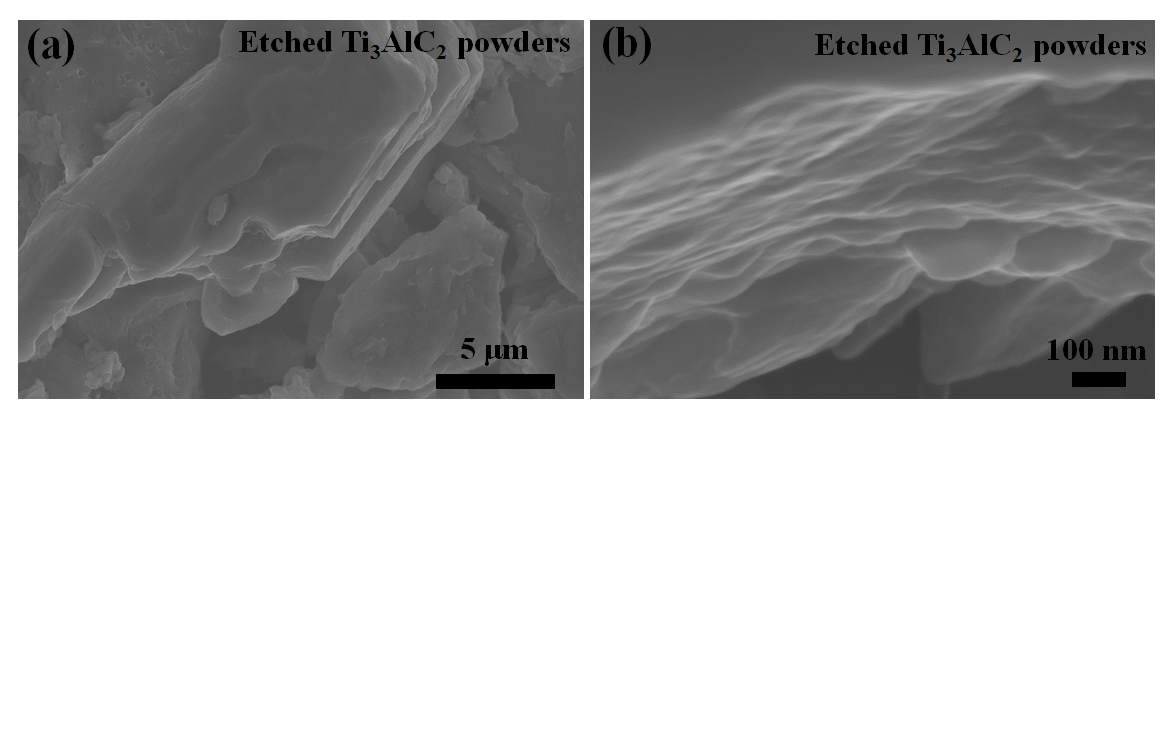


**Figure S2.** SEM images of HF etched Ti_3_AlC_2_ powders with different magnifications.


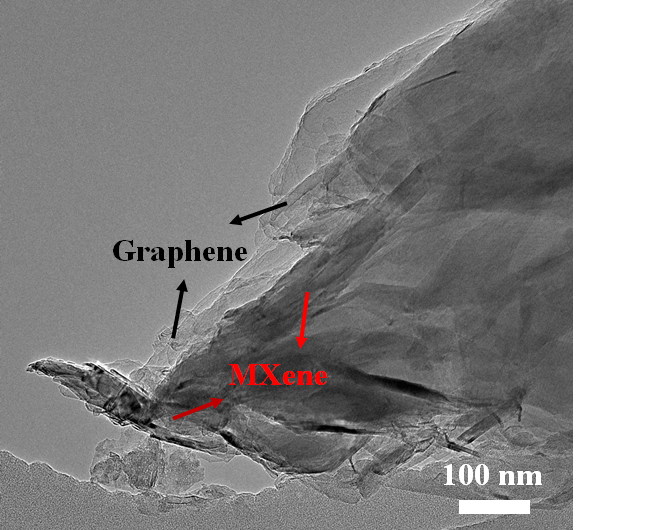


**Figure S3.** Low-magnification TEM image of graphene@MXene hybrid.


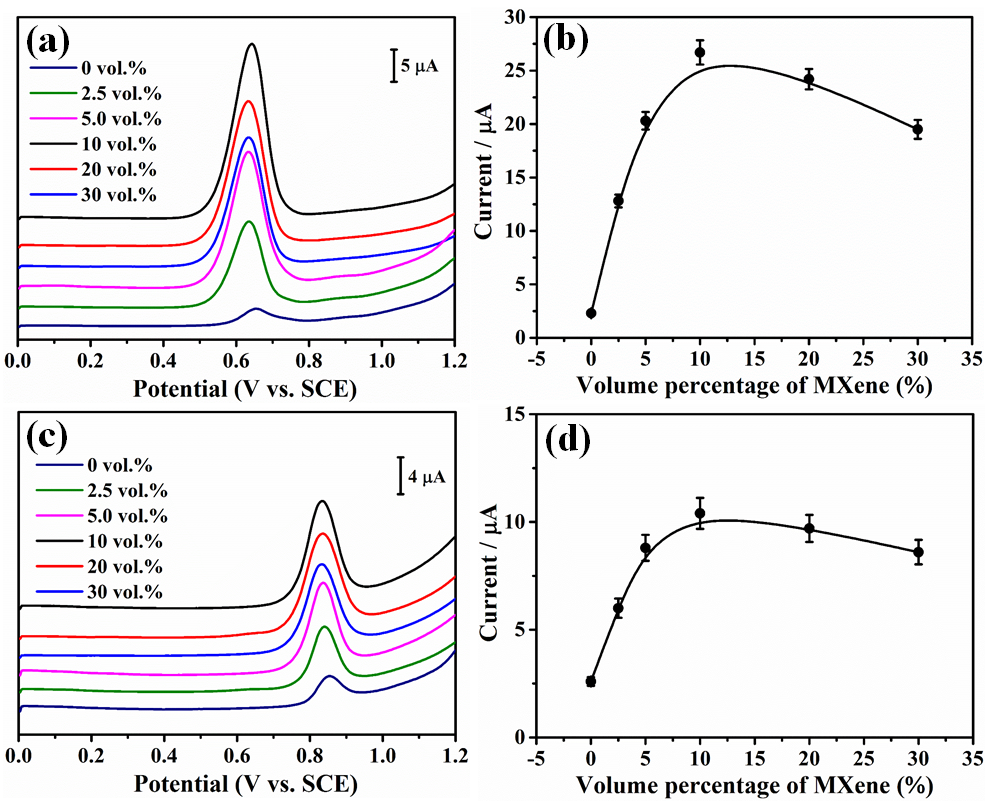


**Figure S4.** Effect of the volume ratio of MXene suspension on the oxidation peak current of (a, b) 1 μM chlorpromazine and (c, d) 1 μM rhodamine B. Accumulation potential: 0 V, accumulation time: 2 min, amount of modifier: 3 μL. Error bar represents the standard deviation of triple measurements.


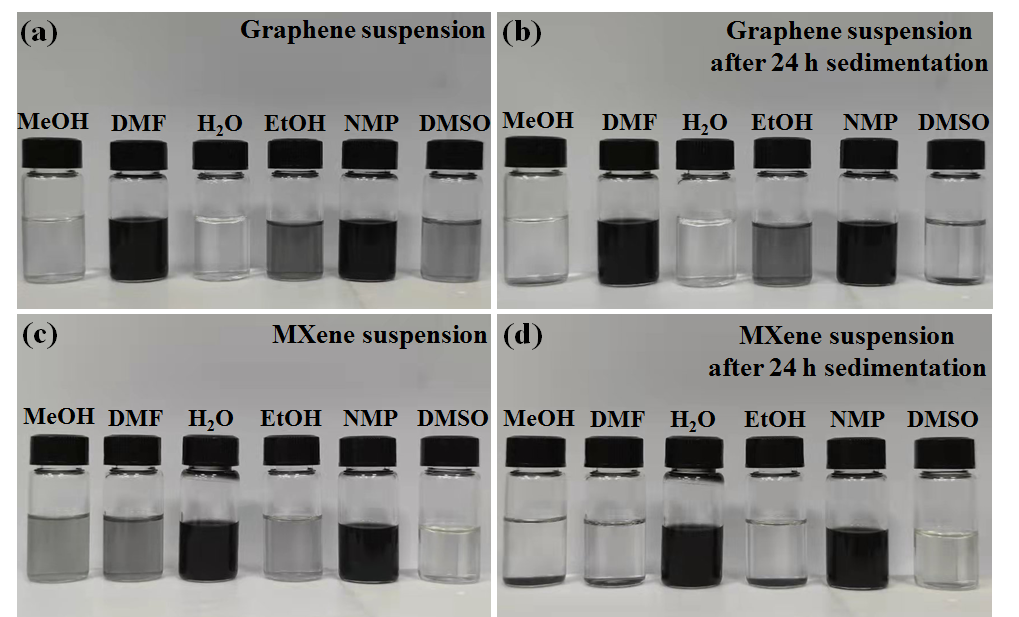


**Figure S5.** Optical images of (a, b) graphene and (c, d) MXene suspensions that prepared in different solvents with and without 24 h sedimentation. Sonication time: 5 h, sonication temperature: 30 ^o^C.


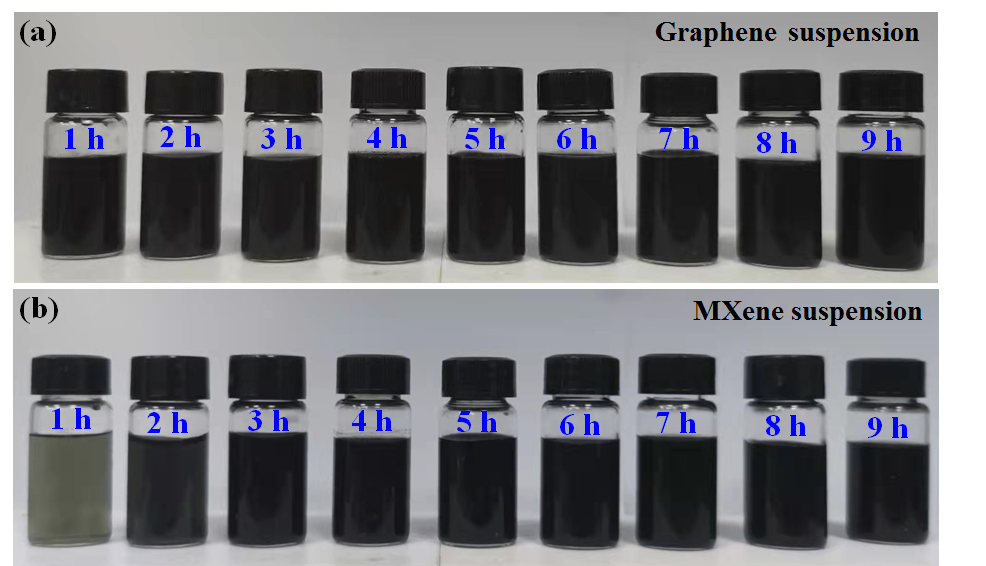


**Figure S6.** Optical images of graphene and MXene suspensions that prepared in NMP with different sonication time. Sonication temperature: 30 ^o^C.


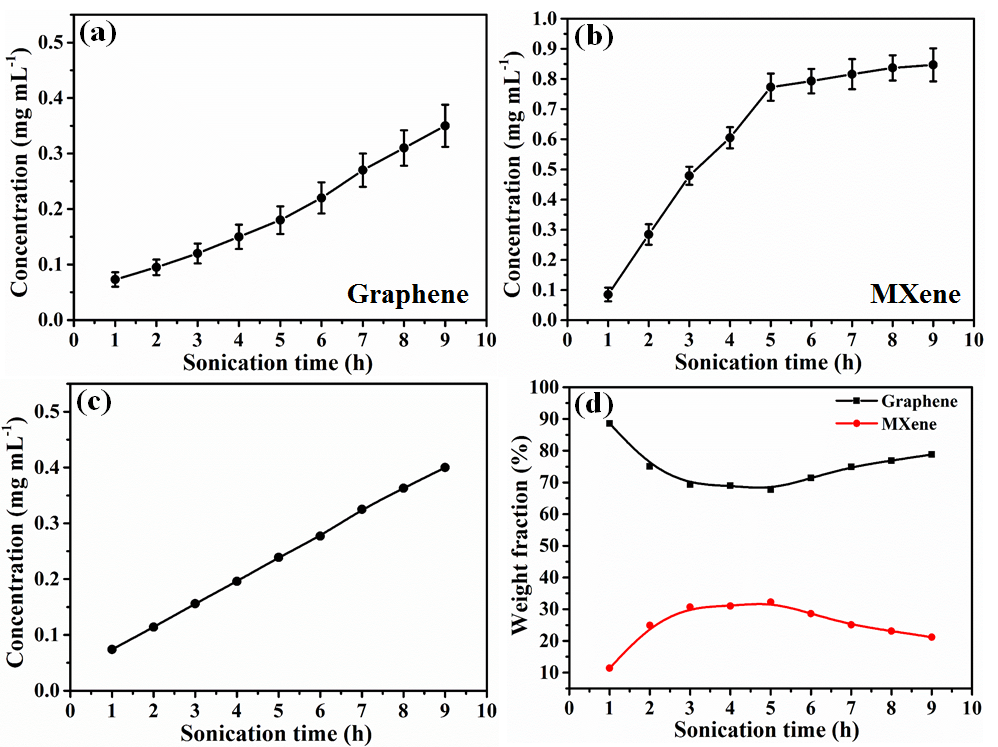


**Figure S7.** Effect of sonication time on the concentration of (a) graphene, (b) MXene and (c) graphene@MXene hybrid suspensions. (d) Variation of weight fraction of graphene and MXene in graphene@MXene hybrid with sonication time. Sonication temperature: 30 ^o^C.


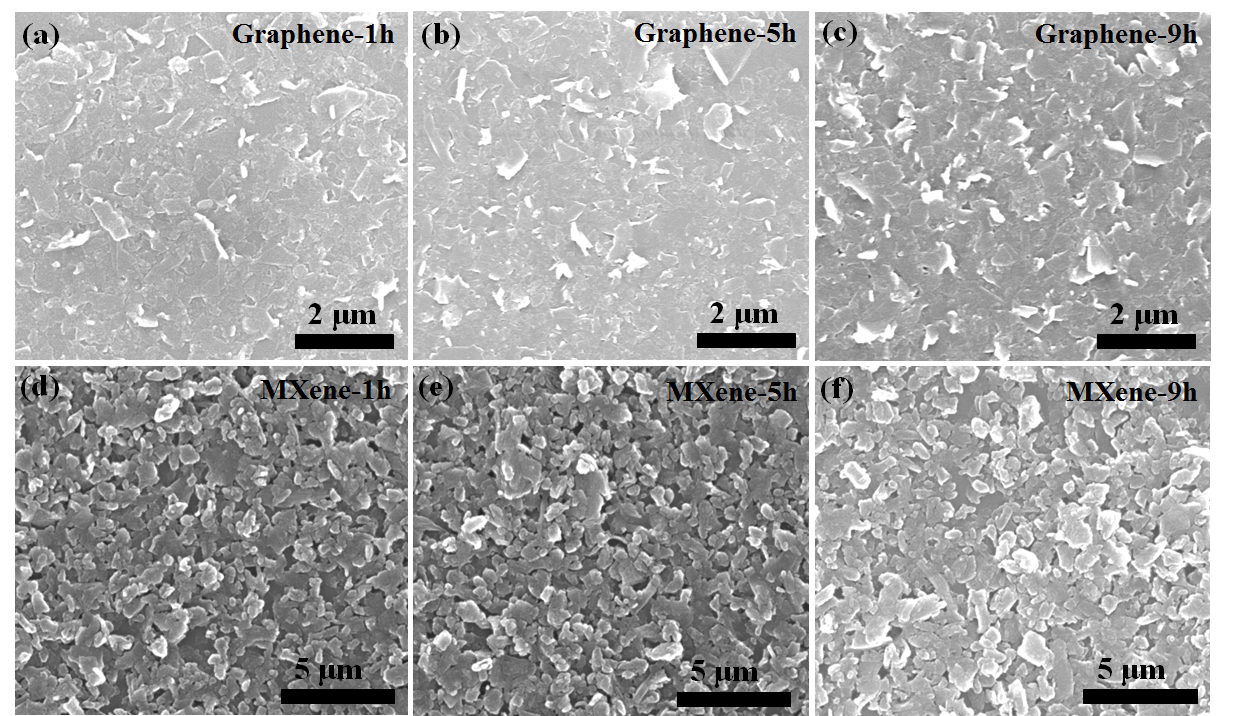


**Figure S8.** SEM images of (a, b, c) graphene and (d, e, f) MXene nanosheets with sonication time of (a, d) 1 h, (b, e) 5 h and (c, f) 9 h in NMP. Sonication temperature: 30 ^o^C.


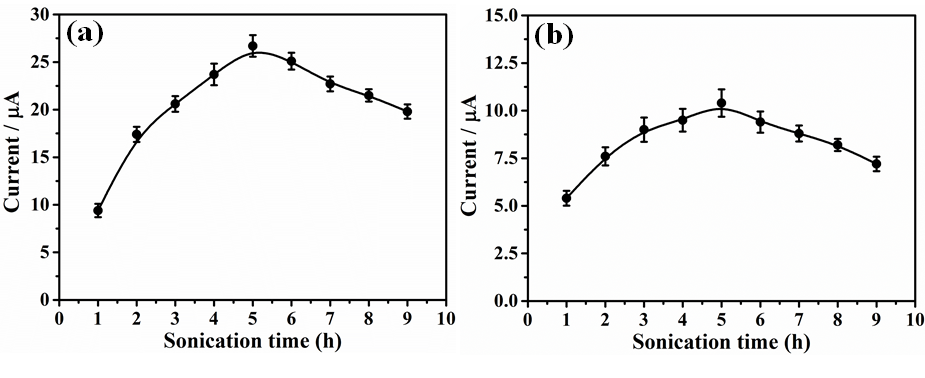


**Figure S9.** Effect of sonication time on the oxidation peak current of (a) 1 μM chlorpromazine and (b) 1 μM rhodamine B at graphene@MXene hybrid modified electrodes. Accumulation potential: 0 V, accumulation time: 2 min, amount of modifier: 3 μL. Error bar represents the standard deviation of triple measurements.


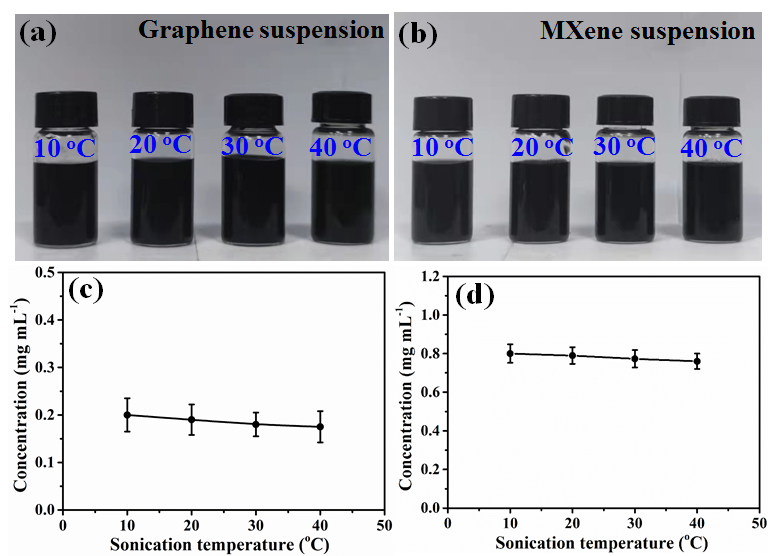


**Figure S10.** Optical images of (a) graphene and (b) MXene suspensions that prepared at different sonication temperature. Effect of sonication temperature on the concentration of (c) graphene and (d) MXene suspensions. Sonication time: 5 h.


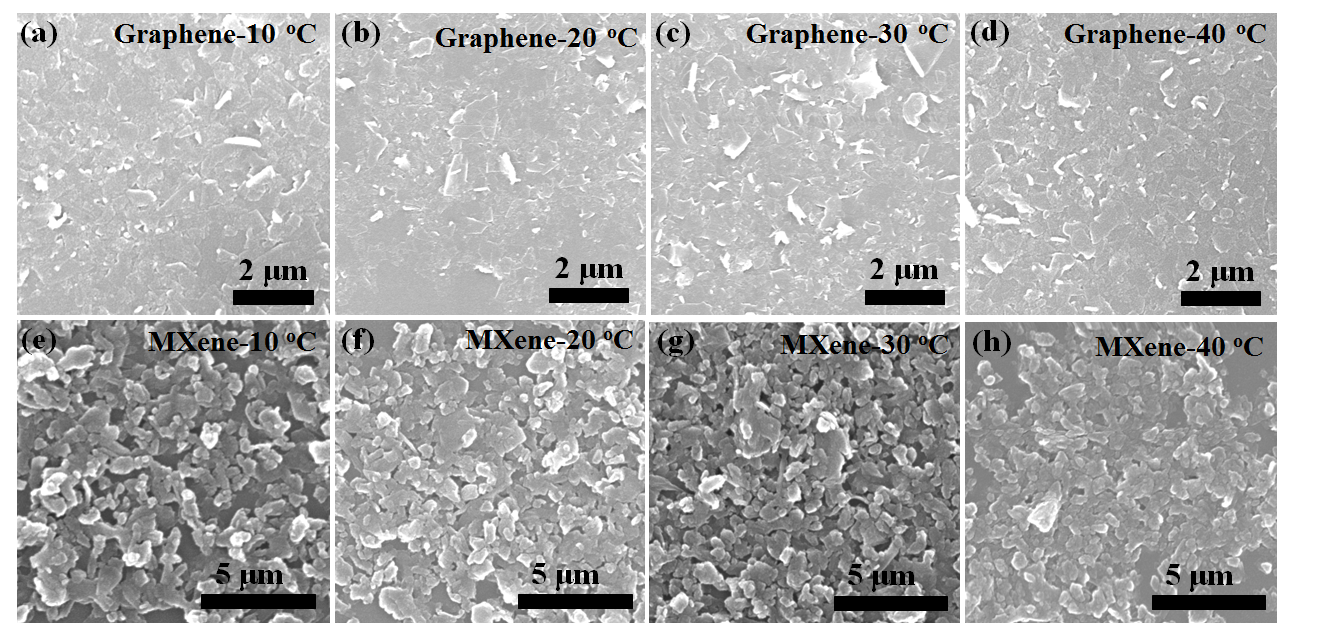


**Figure S11.** SEM images of graphene and MXene nanosheets with different sonication temperature in NMP. Sonication time: 5 h.


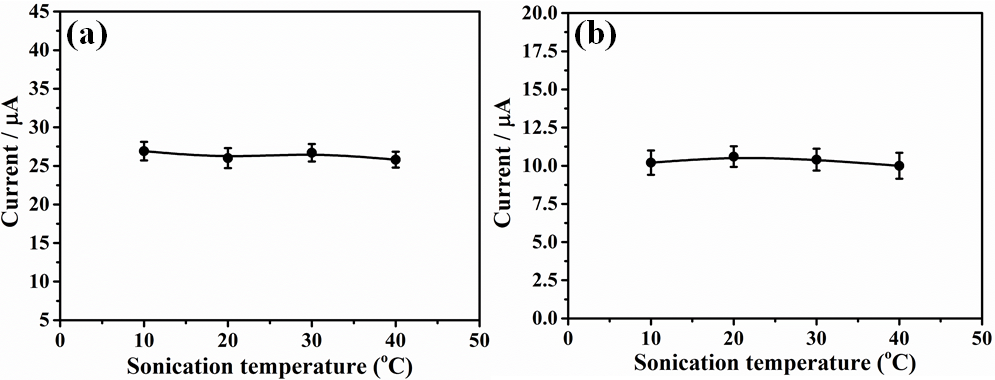


**Figure S12.** Effect of sonication temperature on the oxidation peak current of (a) 1 μM chlorpromazine and (b) 1 μM rhodamine B at graphene@MXene hybrid modified electrodes. Accumulation potential: 0 V, accumulation time: 2 min, amount of modifier: 3 μL. Error bar represents the standard deviation of triple measurements. Sonication time: 5 h.


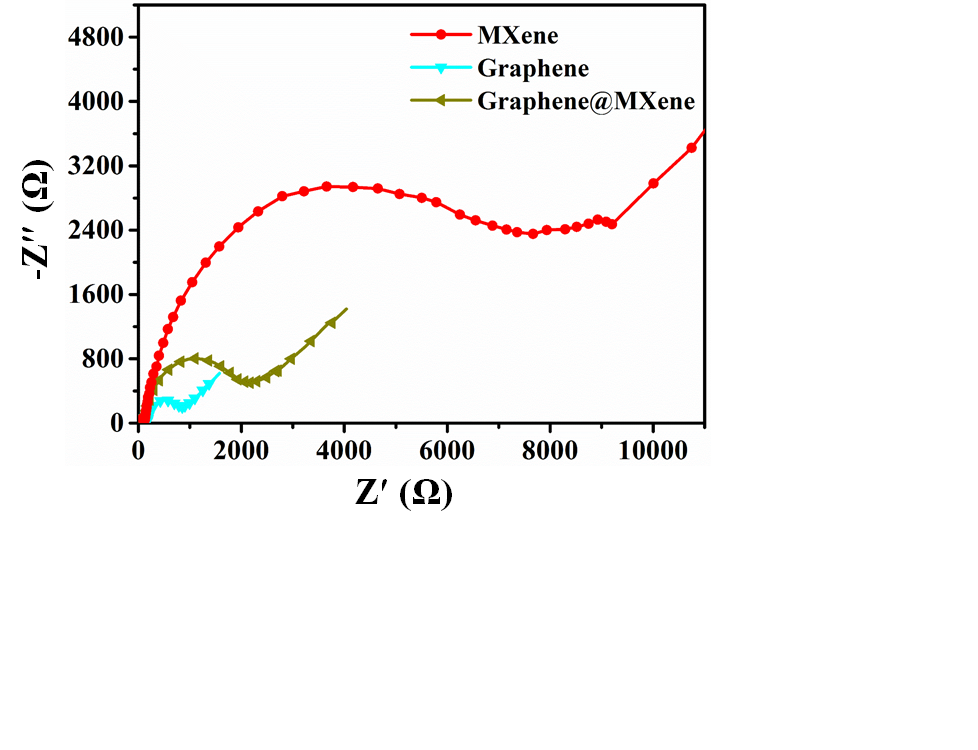


**Figure S13.** Nyquist impedance plots of different electrode materials in 0.1 M, pH 6.5 phosphate buffer solution containing 0.1 M chlorpromazine with applied potential of 0.9 V. Frequency range: 100,000-0.1 Hz; amplitude: 5 mV. Amount of suspension: 3 μL.


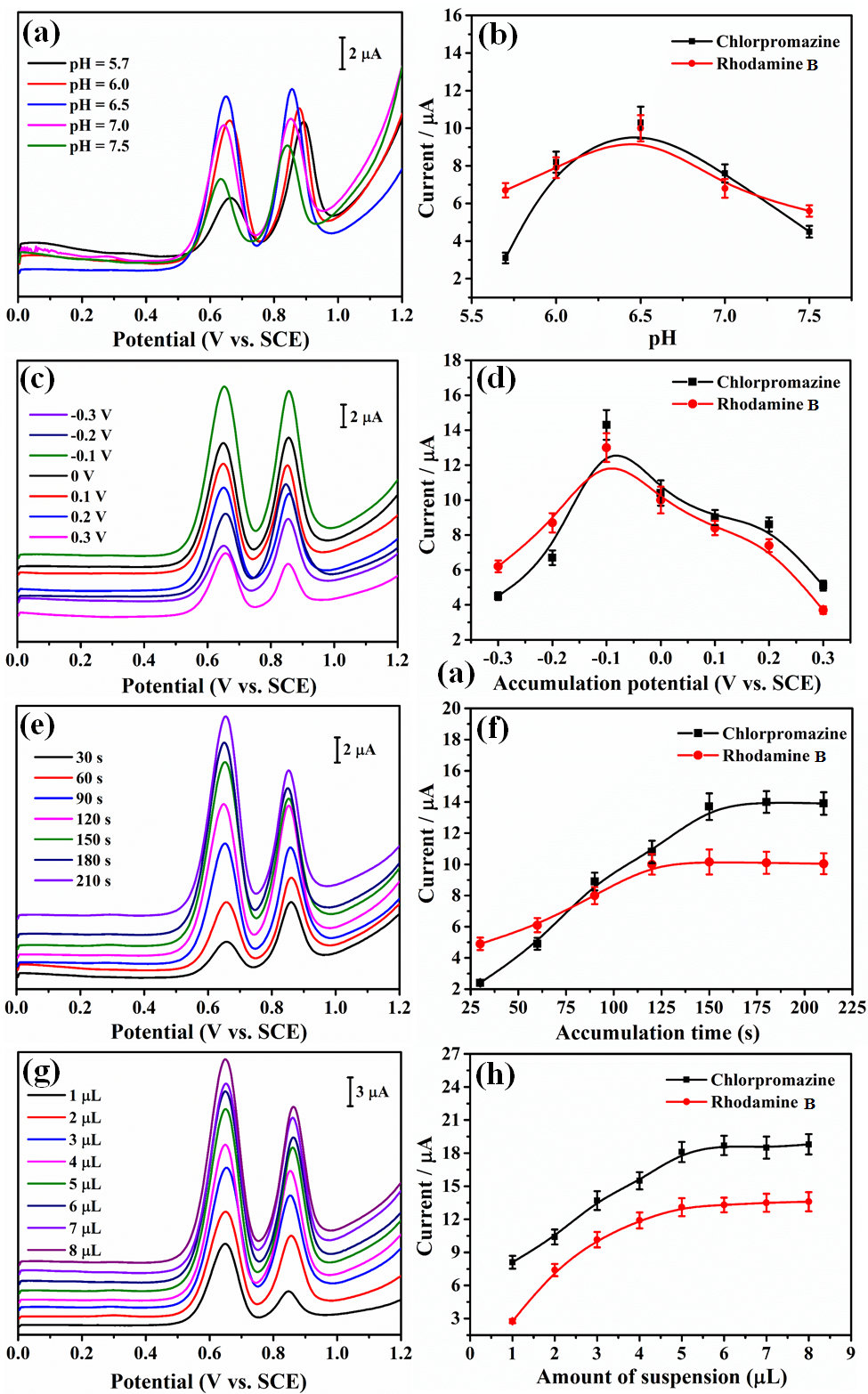


**Figure S14.** Effect of (a-b) pH of electrolyte solution, (c, d) accumulation potential, (e, f) accumulation time and (g, h) volume of suspension on the oxidation peak current of 0.25 μM chlorpromazine and 1 μM rhodamine B at graphene@MXene hybrid modified electrodes. Error bar represents the standard deviation of triple measurements.





**Figure S15.** Stability test of graphene@MXene hybrid modified electrodes in the electrolyte solution. The DPV curves were obtained at the optimal conditions in the presence of 0.25 μM chlorpromazine and 1 μM rhodamine B.

**References**

[S1] H.T. Purushothama, Y.A. Nayaka, M.M. Vinay, P. Manjunatha, R.O. Yathisha, K.V. Basavarajappa, Pencil graphite electrode as an electrochemical sensor for the voltammetric determination of chlorpromazine, J. Sci.-Adv. Mater. Dev. 3 (2018) 161-166.

[S2] B. Unnikrishnan, P.C. Hsu, S.M. Chen, A multipurpose voltammetric sensor for the determination of chlorpromazine in presence of acetaminophen, uric acid, dopamine and ascorbic acid, Int. J. Electrochem. Sci. 7 (2012) 11414-11425.

[S3] D. Bouchta, N. Izaoumen, H. Zejli, M.E. Kaoutit, K.R. Temsamani, A novel electrochemical synthesis of poly-3-methylthiophene-γ-cyclodextrin film: application for the analysis of chlorpromazine and some neurotransmitters, Biosens. Bioelectron. 20 (2005) 2228-2235.

[S4] M.H. Parvin, Graphene paste electrode for detection of chlorpromazine, Electrochem. Commun. 13 (2011) 366-369.

[S5] B.B. Petković, D. Kuzmanović, T. Dimitrijević, M.P. Krstić, D.M. Stanković, Novel strategy for electroanalytical detection of antipsychotic drugs chlorpromazine and thioridazine; possibilities for simultaneous determination, Int. J. Electrochem. Sci. 12 (2017) 3709-3720.

[S6] N. Sebastian, W.C. Yu, Y.C. Hu, D. Balram, Y.H. Yu, Sonochemical synthesis of iron-graphene oxide/honeycomb-like ZnO ternary nanohybrids for sensitive electrochemical detection of antipsychotic drug chlorpromazine, Ultrason. Sonochem. 59 (2019) 104696.

[S7] M.H. Parvin, M.B. Golivand, M. Najafi, S.M. Shariaty, Carbon paste electrode modified with cobalt nanoparticles and its application to the electrocatalytic determination of chlorpromazine, J. Electroanal. Chem. 683 (2012) 31-36.

[S8] A.E. Kartika, H. Setiyanto, R.V. Manurung, S.N.A. Jenie, V. Saraswaty, Silver nanoparticles coupled with graphene nanoplatelets modified screen-printed carbon electrodes for rhodamine B detection in food products, ACS Omega 6 (2021) 31477-31484.

[S9] Q.G. He, J. Liu, Y.L. Tian, Y.Y. Wu, F. Magesa, P.H. Deng, G.L. Li, Facile preparation of Cu_2_O nanoparticles and reduced graphene oxide nanocomposite for electrochemical sensing of rhodamine B, Nanomaterials 9 (2019) 958.

[S10] J.Y. Sun, T. Gan, Y. Li, Z.X. Shi, Y.M. Liu, Rapid and sensitive strategy for rhodamine B detection using a novel electrochemical platform based on core-shell structured Cu@carbon sphere nanohybrid, J. Electroanal. Chem. 724 (2014) 87-94.

[S11] P.H. Deng, J.Y. Xiao, J.X. Chen, J.X. Feng, Y.P. Wei, J.S. Zuo, J. Liu, J.H. Li, Q.G. He, Polyethylenimine-carbon nanotubes composite as an electrochemical sensing platform for sensitive and selective detection of toxic rhodamine B in soft drinks and chilli-containing products, J. Food Compos. Anal. 107 (2022) 104386.

[S12] M. Golestaneh, S.M. Ghoreishi, Sensitive determination of rhodamine B in real samples at the surface of a multi-walled carbon nanotubes paste electrode, Anal. Bioanal. Electro. 12 (2020) 81-92.

[S13] S.X. Feng, W.G. Ding, Y. Zhang, J.W. Wu, Zou, Z.M. T.F. Wu, Q. Tang, A nanoscale Nd-based metal-organic framework electrochemical sensor for rapid detection of Rhodamine B, J. Solid State Chem. 303 (2021) 122508.

[S14] Q.G. He, J. Liu, Y.H. Xia, D. Tuo, P.H. Deng, Y.L. Tian, Y.Y. Wu, G.L. Li, D.C. Chen, Rapid and sensitive voltammetric detection of rhodamine B in chili-containing foodstuffs using MnO_2_ nanorods/electro-reduced graphene oxide composite, J. Electrochem. Soc. 166 (2019) B805-B813.
